# A lipidomic approach to the study of human CD4^+^ T lymphocytes in multiple sclerosis

**DOI:** 10.1186/s12868-015-0183-1

**Published:** 2015-07-24

**Authors:** Daniele Vergara, Michele D’Alessandro, Antonia Rizzello, Lidia De Riccardis, Paola Lunetti, Piero Del Boccio, Francesca De Robertis, Giorgio Trianni, Michele Maffia, Anna M Giudetti

**Affiliations:** Department of Biological and Environmental Sciences and Technologies, University of Salento, via Monteroni, Lecce, Italy; Laboratory of Clinical Proteomic, “Giovanni Paolo II” Hospital, ASL-Lecce, Piazzetta F. Muratore, Lecce, Italy; Department of Medical, Oral and Biotechnological Sciences, Research Centre on Aging (Ce.S.I), “G. d’Annunzio” University Foundation, Chieti-Pescara, Italy; Department of Neurology, “Vito Fazzi” Hospital, ASL-Lecce, Piazzetta F. Muratore, Lecce, Italy

**Keywords:** Cardiolipin, CD4^+^ T lymphocyte, Lipidomics, MALDI-TOF, Multiple sclerosis, 9-aminoacridine

## Abstract

**Background:**

Lipids play different important roles in central nervous system so that dysregulation of lipid pathways has been implicated in a growing number of neurodegenerative disorders including multiple sclerosis (MS). MS is the most prevalent autoimmune disorder of the central nervous system, with neurological symptoms caused by inflammation and demyelination. In this study, a lipidomic analysis was performed for the rapid profile of CD4^+^ T lymphocytes from MS patient and control samples in an untargeted approach.

**Methods:**

A matrix-assisted laser desorption/ionization-time of flight (MALDI-TOF) mass spectrometry based approach was used for the analysis of lipid extracts using 9-aminoacridine as matrix. Lipids were analyzed in negative mode and selected species fragmented using MALDI tandem mass spectrometry for their structural assignments.

**Results:**

The analysis reveals some modifications in the phospholipid pattern of MS CD4^+^ T lymphocytes with respect to healthy controls with a significant increase of cardiolipin species in MS samples.

**Conclusions:**

These results demonstrate the feasibility of a MALDI-TOF approach for the analysis of CD4^+^ lipid extracts and suggest how alterations in the lipid metabolism characterized lymphocytes of MS patients.

## Background

Multiple sclerosis (MS) is a demyelinating inflammatory disease of the central nervous system (CNS) with heterogeneous clinical outcomes [1]. The causes of MS are not well understood but there is compelling evidence that a combination of factors such as environment, viruses and dietary conditions, in conjunction with genetic susceptibility might drive an autoimmune response against structure of CNS. Autoimmune mechanisms of CNS damage are primarily mediated by auto-reactive CD4^+^ T cells, which are specific for encephalitogenic epitopes of myelin peptides [2].

Migration of autoimmune T cells from the periphery into CNS parenchyma leads to inflammation, demyelization and damage of axons, oligodendrocytes and neurons [1]. This autoimmune T cell mediated tissue damage results in an impairment of motor function leading to paralysis. Disease is progressive and often takes a relapsing-remitting course. Progression and severity of the disease as well as types of CNS lesions are highly heterogeneous among patients with MS [2].

T lymphocytes play a central role in the pathogenesis of MS [2] and many successfully therapies in MS have been used T cell target approaches [3]. T cells are found in all four of the described histopathologic subtypes of MS [4]. Both CD4^+^ T and CD8^+^ T cells have been demonstrated in MS lesions, with CD4^+^ T cells predominating in acute lesions and CD8^+^ T cells being observed more frequently in chronic lesions [4]. It has been reported that CD4^+^ T lymphocytes MHC class II-restricted, mainly polarized as Th1 cells induced CNS inflammation by producing inflammatory cytokines such as IFN-γ, IL-2, TNF-α and lymphotoxin [5]. Activated myelin-reactive CD4^+^ T cells are present in the blood and cerebrospinal fluid of MS patients [4].

A main feature of MS is the change in the trafficking properties of immune cells throughout the blood–brain barrier [6]. CD4^+^ cells, positive for PSGL-1 surface antigen, were found to transmigrate throughout the blood–brain barrier to levels significantly higher than the equivalent population isolated from healthy subjects [7]. Moreover, inhibiting leukocyte adhesion to blood–brain barrier by antibodies against α4-integrin reduced the number of lesion in CNS of MS patients [8]. Regardless of the complexities associated with MS pathogenesis, the infiltration of immune cells and their activities likely contribute to the loss of myelin and axonal degradation that accompanies progression of the disease. Therefore, it is critical to understand the mechanisms involved in the transmigration of leukocytes into regions of the CNS where they are normally excluded.

Membrane lipid composition plays an important role in the dynamic of cells. In particular, changes in membrane lipid composition that alter membrane fluidity may induce modifications in the functions of proteins and receptors involved in signaling pathways. Immune cell membrane lipids are involved in many functions and fatty acid abnormalities have been reported in immune cells from patients with MS [9]. Studies have provided that a specific lipid modification by peroxidative mechanisms could be a significant pathogenic factor in MS [10]. Moreover changes in lipid composition of cerebral fluid and plasma, in MS, have been demonstrated [11].

Recently, developments in lipid mass spectrometry provided the ability to describe changes in lipid compositions that occur with disease progression or after a specific treatment, giving also the opportunity to identify lipid biomarkers and clarify cellular metabolism of individual lipid species [12].

In the present study, we applied a mass spectrometry-based lipidomics profiling to identify molecular lipid species associated with MS. To perform this, the lipid composition of human CD4^+^ T cells isolated from a cohort of MS patients and normal subjects was screened by Matrix-Assisted Laser Desorption Ionization Time-of-Flight/Time-of-Flight (MALDI-TOF/TOF) analysis. Multivariate statistical analysis revealed that significant differences of specific lipid species characterize MS patients compared with normal subjects.

## Methods

### Ethical permission

The study was approved by the Vito Fazzi Hospital Ethics Committee (ASL_LE-General Manager Resolution n 228, February 11, 2014). Informed consent was obtained from each patient prior to entry into the study, according to the declaration of Helsinki.

### Participants/study population

Height consenting patients suffering from defined relapsing-remitting type of MS (RR-MS) with an age range of 18–40 years, were clinically diagnosed at the Vito Fazzi Hospital in Lecce, and included into the study. Patients had a definite diagnosis of MS, as defined by the revised McDonald Criteria [13], and were classified as having relapsing remitting MS (RRMS) according to the Lublin-Rheingold classification [14]. For the evaluation of disease severity the Expanded Disability Status Scale (EDSS) was used [15]. The characteristics of both MS and healthy donors are reported in the Table 1. None of the patients was on treatment with interferon, steroids or other immunosuppressive drugs for at least 3 months prior to entering the study. For each patient, blood samples were obtained at baseline; samples were also collected from a number of 5 healthy controls with an age range of 28–48 years and used for the following analysis.

**Table 1:** Clinical characteristics for the multiple sclerosis (MS) and healthy control (HC) groups

No.	Sex	Age (years)	Diagnosis/classification	EDSS
MS_1_	M	35	RRMS	1.5
MS_2_	F	32	RRMS	3.5
MS_3_	F	27	RRMS	2.5
MS_4_	F	18	RRMS	3.0
MS_5_	F	35	RRMS	1.7
MS_6_	F	24	RRMS	3.9
MS_7_	M	35	RRMS	2.0
MS_8_	M	40	RRMS	2.3
HC_1_	M	37	Healthy control	na
HC_2_	F	28	Healthy control	na
HC_3_	F	30	Healthy control	na
HC_4_	M	48	Healthy control	na
HC_5_	F	32	Healthy control	na

*RRMS* relapsing remitting MS, *EDSS* Expanded Disability Status Scale, *na* not applicable.

### Chemicals

Solvents were reagent grade and purchased from Baker. 9-aminoacridine (9-AA) matrix and cardiolipin standard (from bovine heart) were purchased from Sigma. Ficoll-Paque PLUS was GE healthcare life sciences. CD4^+^ T Cell Isolation Kit was from Miltenyi Biotec (Bergisch Gladbach, Germany).

### Cell isolation

Venous blood from consenting participants was collected into anti-coagulant EDTA tubes (Beckman Coulter, South Africa). Plasma samples were obtained from patients with MS and from healthy controls (HC). PBMC fractions were isolated from whole blood using Ficoll-Paque density-gradient centrifugation as described [16]. CD4^+^ T cells were purified by negative selection using an indirect magnetic cell sorting kit.

### Lipid extraction from CD4^+^ T lymphocytes

The extraction of lipids from CD4^+^ T lymphocytes was performed using the method of Bligh and Dyer [17]. Briefly, to 20 μl (about 30 μg of protein) of cell suspension was added a solution of chloroform/methanol (1:2, v/v) and, after vigorous stirring, the final mixture was kept at 4°C and then centrifuged at 19,800×*g* for 5 min. To the supernatant obtained after centrifugation was added chloroform and a 5% (w/v) solution of NaCl. The mixture was stirred and placed at 4 °C for a time sufficient to obtain the formation of two phases, the lower phase (chloroform) containing lipids and the upper aqueous phase (water and methanol). The chloroform phase was then collected, filtered through nylon filters, brought to dryness under a stream of nitrogen and then resuspended in an appropriate volume of chloroform for the subsequent analyses.

### Fatty acid analysis

Fatty acid composition of CD4^+^ T cells was determined processing the samples as in [18]. Briefly, 20 μl of cell suspension (about 30 μg protein) was subjected to saponification for 90 min at 85–90°C using an alcoholic solution of KOH. After acidification of the mixture, the fatty acids were extracted with petroleum ether. The methyl esters of fatty acids (FAME) were prepared by heating the extract at 65°C for 30 min with a solution of boron trifluoride in methanol (17% BF3) and then analyzed by gas-chromatography. The helium carrier gas was used at a flow rate of 1 ml min^−1^. FAME were separated on a 30 m × 0.32 mm HP5 (Hewlett Packard) capillary column. The injector and detector temperature was maintained at 250°C. The column was operated isothermally at 150°C for 4 min, and then programmed to 250°C at 4°C/min. Peak identification was performed by using known standards and relative quantitation was automatically carried out by peak integration.

### MALDI-TOF/TOF analysis

Lipids mass spectra were acquired in positive and negative ion reflector mode (detection range: 500–2,000 mass/charge, *m*/*z*), using a Bruker Daltonics Ultraflex Extreme MALDI-TOF/TOF mass spectrometer. Samples were analyzed using 9-aminoacridine (9-AA) as a MALDI matrix. Matrix solution was prepared by dissolving 10 mg 9-AA in 2-propanol:acetonitrile (60:40 v/v) (10 mg/mL) as reported [19]. Lipids, dissolved in the same matrix solvent, were mixed 1:1 with matrix and spotted on a MTP AnchorChip 384 target plate for mass analysis. Samples were allowed to air dry before insertion into the MALDI-TOF analyzer. Before each data acquisition, an external calibration was conducted using the peptide standard calibration kit mixture (Bruker Daltonics). For MALDI-MS and MS/MS analysis, ions from 2000 consecutive laser shots were collected under reflectron mode and summed into one spectrum. The collected spectra were then processed with FlexAnalysis 3.4 (Bruker Daltonics). A specific lipid database (Lipid Maps Database, http://www.lipidmaps.org) was used to facilitate and confirm the assignment of phospholipid species.

### Statistical analysis

The data matrix was exported for partial least squares discriminant analysis (PLS-DA) using Simca-P+ 11.0 software (Umetrics AB, Umeå, Sweden). Raw data were prepared to PLS-DA analysis through unit variance scaling (UV-scaling) and mean centering as default pre-processing operations. To searching for the variables that have the greatest influence in class discrimination we used the Variable Importance Analysis in SIMCA-P+. The software indicates that terms with large Variable Importance in the Projection (VIP) value, larger than 1, are the most relevant for group’s discrimination. The major discriminant variables were selected and the possibly outlier values were searched by the online tool: outlier calculator (GraphPad Software) that performs Grubbs’ test and setting a significance level of 95% (alpha equal to 0.05). Subsequently, D’Agostino and Pearson omnibus normality test was performed in order to determine the normality of each variable measured in each group. When normality was accepted the Student’s t-test was employed, otherwise the Mann–Whitney U-test was used for comparing the groups. GraphPad Prism was used to perform all these univariate analyses (GraphPad software, Inc. USA). Finally, the differential significant variables were identified using Lipidmaps Database.

## Results

### MALDI-TOF analysis and identification of lipids

**Figure 1:**
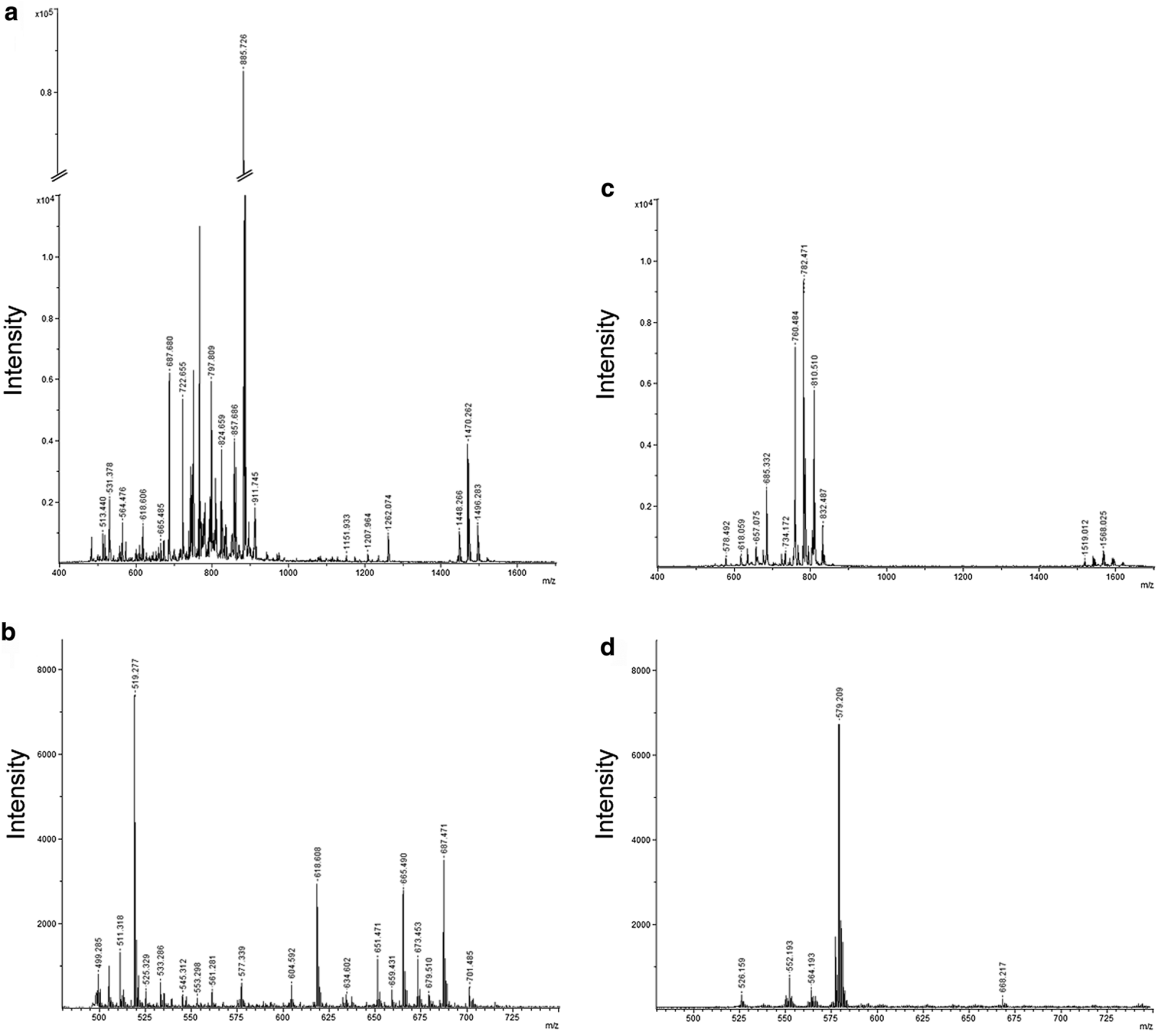
MALDI-TOF mass spectra of lipids from CD4^+^ T lymphocytes obtained in positive and negative mode. Representative MALDI-TOF mass spectra of lipids extracted from CD4^+^ T lymphocytes (*upper panels*) prepared by the Bligh and Dyer procedure. Spectra were acquired in both negative (**a**) and positive (**b**) mode, using the 9-AA matrix by averaging 2.000 consecutive laser shots. MALDI-TOF spectra of 9-AA matrix detected in negative (**c**) and positive mode (**d**) is reported. Spectra were acquired using a Ultraflex Extreme MALDI-TOF/TOF (Bruker).

Figure 1 shows the representative mass spectrum of total lipid extracts prepared from CD4^+^ T lymphocytes using 9-AA as matrix. Lipids are charged molecules and due to presence of different charged groups, mass spectrometry analysis of these molecules can be performed in both positive and negative ion mode. However, the process of ionization is not only dependent on lipid charge but also influenced by different factors including the matrix used for MALDI analysis [20, 21]. For this reason, lipids extracts were analyzed in both negative and positive mode and results are shown in Figure 1a, b, respectively.

In the lower panel (Figure 1c, d) peaks corresponding to 9-AA alone, in positive and negative mode, are also showed. Their identification allowed excluding matrix peaks from the analysis of our lipid samples.

Although the number of matrix peaks detected was higher for 9-AA observed in negative mode, this ionization was found to be more efficient for the analysis of CD4^+^ lipids. In fact, if we compare the intensity of signals of the two spectra reported in Figure 1a, b, ionization in negative mode provided higher quality spectrum compared to the positive one. The signal intensity and the number of peaks detected were both higher. These results correlate with previous studies reporting an increase in signal intensity in negative ion mode when using 9-AA matrix [20].

**Figure 2:**
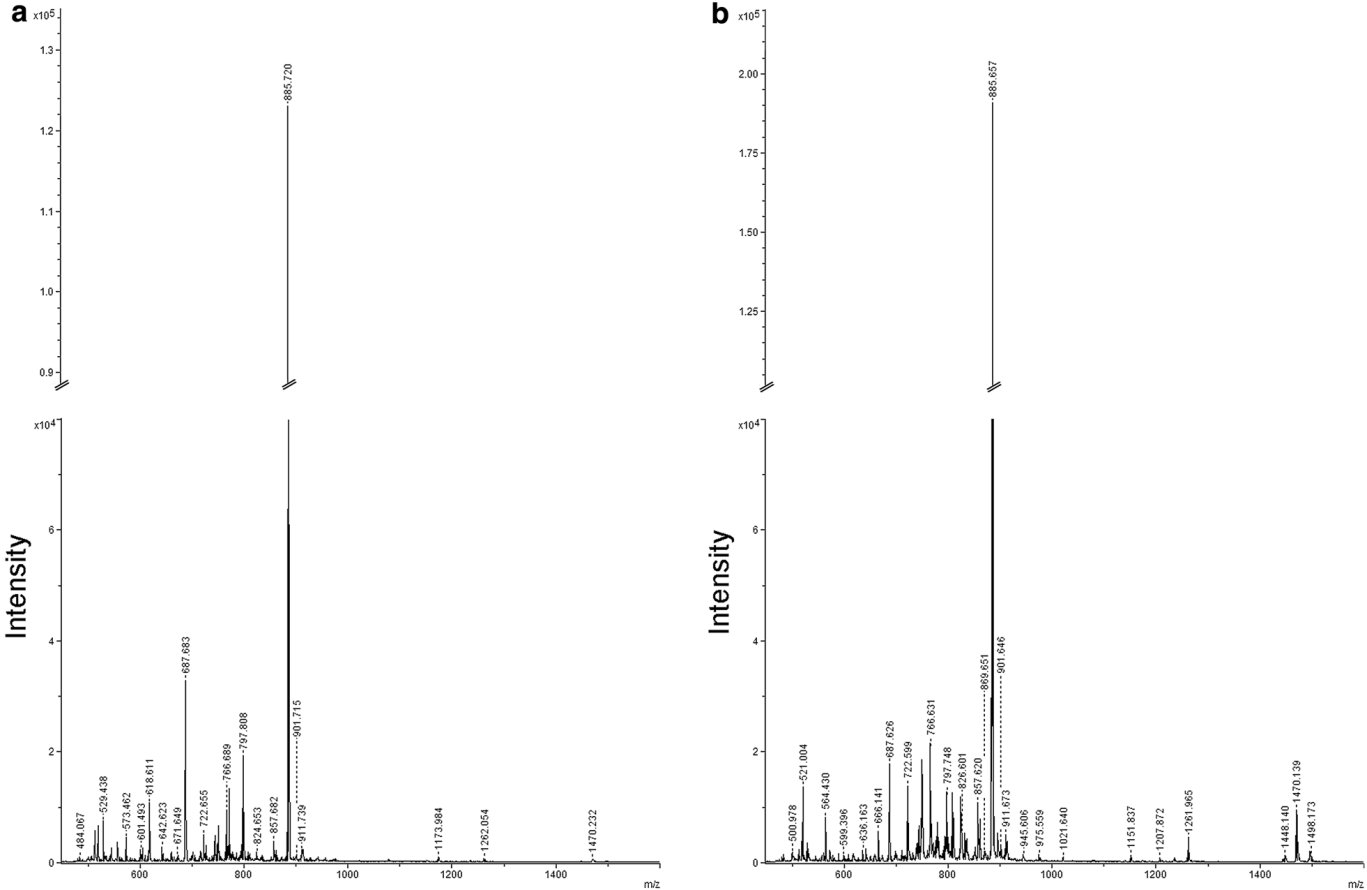
MALDI-TOF mass spectra of CD4^+^ lipids from MS and control subjects obtained in negative mode. Representative negative ion MALDI-TOF spectrum of total lipid extracts from healthy controls (**a**) and multiple sclerosis patients (**b**) CD4^+^ T lymphocytes, using the 9-AA matrix by averaging 2.000 consecutive laser shots.

These observations demonstrated that better results were obtained using 9-AA in negative mode, and these experimental conditions were preferred for the analysis of our samples. In Figure 2, spectra from healthy (Figure 2a) and MS (Figure 2b) subjects obtained with 9-AA in negative mode are reported. As showed, overall lipid pattern appears very similar, dominated by signals in the range of 600–1,500 *m*/*z* compatible with phospholipid species [21] as also identified by post-spectral processing (Table 2).

**Table 2:** Assignment based on *m/z* measurements of peaks detected in the negative ion mass spectra of lipids from CD4^+^ T lymphocytes using 9-AA as matrix

*m*/*z* value	Assignment [M-H]^−^	*m*/*z* value	Assignment [M-H]^−^
513,400	Matrix	799,767	SM d17:1/24:0 or d18:1/23:0
529,389	Matrix	800,596	PE 41:0 or PS 37:2
573,412	Matrix	802,607	PE 42:6 or PS 38:1
617,430	Matrix	806,584	PE 42:4
618,557	Matrix	808,601	PE 41:4 or PS 38:5
619,371	PA 30:0	826,604	PE 42:2
666,141	Matrix	828,631	PE 42:1
687,632	DG 41:3	836,632	PE 43.4 or PS 40:5
722,603	PE 36:4	850,617	PE 44:4 or PS 42:4
738,599	Sphyngolipid 15:2/22:0	852,631	PE 44:3
742,634	PE 36:2	857,625	PG 42:2
743,696	SM 37:1	859,642	PI 36:2
744,652	PE 36:1	861,640	PG 42:0 or PI 36:2
746,600	PE 36:0	883,642	PI 38:5
750,636	Sphyngolipid 18:2/20:1	885,661	PG 44:2
760,934	PE O-38:0	901,657	PI 40:2
764,619	PE 38:5	909,663	PI 40:6
766,634	Sphyngolipid 18:2/21:0	911,618	PI 40:6
769,716	PA 42:1 or SM 39:0	913,696	PI 40:4
771,732	PA 42:0 or SM 38:1	943,623	PI 42:3
776,849	PE O-40:6 or PS O-36:0	1.448,140	CL 72:8
778,651	PE 40:4	1.470,150	CL 74:11
780,560	PE 41:4	1.472,170	CL 74:10
782,562	PE 41:3	1.494,150	CL 75:6
792,650	PE 40:5	1.496,170	CL 75:5
794,675	Sphingolipid 18:2/23:0	1.498,190	CL 75:4
795,736	PI O-32:0		
797,754	SM d17/24:1		

For the identification, mass tolerance was set to ±0.1.

*CL* cardiolipin, *DG* diacylglycerol, *PA* phosphatic acid, *PE* phosphatidylethanolamine, *PS* phosphatidylserine, *PI* phosphatidylinositol, *PG* phosphatidylglycerol, *SM* sphingomyelin.

### Multivariate analysis of lipid species

**Figure 3:**
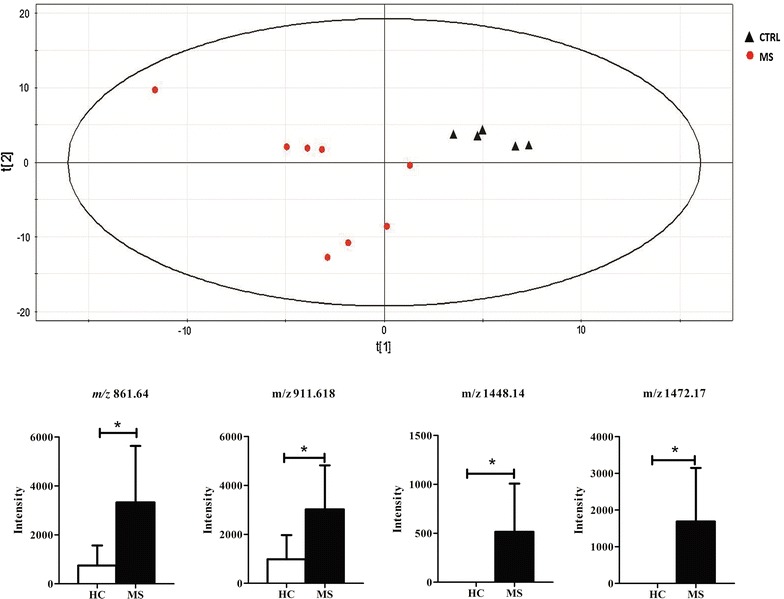
Partial least squares discriminat analysis. Partial least squares discriminat analysis (PLS-DA) score plot from lipidomics data derived from control (*black triangle*) and MS (*red circle*) patients. Below, *histograms* show peaks that were significantly different (P < 0.05) among groups. Data reported as mean ± SD.

The signals of the MALDI spectra were exported in a final matrix in a compatible format for the multivariate analysis with Simca-P+ software. In this matrix all molecules were reported as variables identified by its own *m*/*z* value related to each observation (patient). To obtain the maximum separation between the two clinical groups, all variables were processed by Partial Last Squares Discriminant Analysis (PLS-DA). Figure 3a shows the PLS-DA score plot of the first two principal components. The graph highlights the separation tendency between the MS (in red) and healthy (in black) groups based on their lipid profile. One MS sample (MS_1_) was dispersed among control subjects probably due to its low EDSS score (Table 1).

Interpreting a PLS-DA model with a lot of components and a multitude of response factors can be a complex task. In this model the most influential molecules responsible for the separation between classes are variables that have the greatest influence in PLS-DA. Simca-P+ software generates a list of all metabolite analyzed, sorted by a score number named VIP. The VIP score reflects the variable’s contribution on the classification, and so can be used to discover the most relevant differential metabolites for the group separation. Lipid molecules with a VIP score greater than 1 were taken in consideration to evaluate their significance in the univariate statistical analysis by comparing the relative intensity of each lipid molecules through a t-test or Mann–Whitney test between the two clinical groups in order to confirm their ability to discriminate MS patients from healthy controls. Four metabolites showed a significant p-value (p-value <0.05) between MS patients and healthy controls: *m*/*z* 861,640 that, on the basis of our assignment (Table 2), corresponded to two possible molecular species namely phosphatidylglycerol or phosphatidylinositol (respectively PG 42:0 or PI 36:2); *m*/*z* 911,618 identified as phosphatidylinositol (PI 40:6); *m*/*z* 1,448,140 and *m*/*z* 1,472,170 identified as cardiolipins (respectively as CL 72:8 and CL 74:10). Due to the presence of various isomers, some identified molecular species were classified only on the basis of their class, the total number of the carbons in lateral chains and the total number of double bonds (unsaturation). These results are summarized in the Table 3 while the histogram in the Figure 3 shows the intensity of each differential expressed metabolite between the two clinical groups.

**Table 3:** Metabolites whcich allowed the separation between healthy and multiple sclerosis patients selected on the the basis of VIP

*m/z*	Regulation in MS	P value	Ion	Lipid species
861,640	Up-regulated	0.037	[M-H]^−^	PG 42:0 or PI 36:2
911,618	Up-regulated	0.042	[M-H]^−^	PI 40:5
1.448,140	Up-regulated	0.042	[M-H]^−^	CL 72:8
1.472,170	Up-regulated	0.047	[M-H]^−^	CL 74:10

A P value <0.05 was set as the threshold to define significant differences.

### MS/MS spectra of cardiolipin

**Figure 4:**
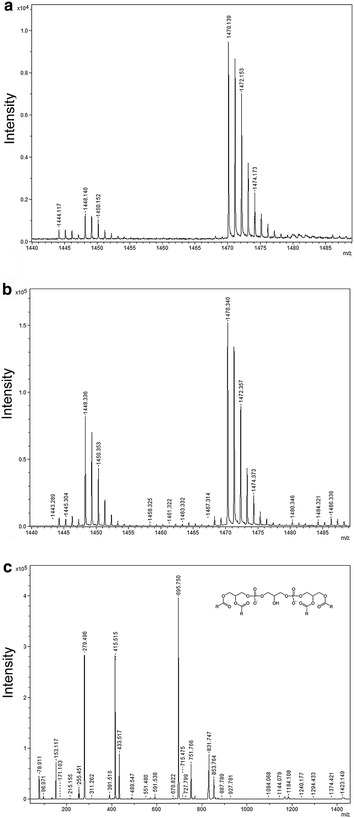
MS/MS based characterization of the cardiolipin *m*/z 1,448,14 ion. Representative MS spectra of cardiolipin species obtained from a MS sample (**a**), and from cardiolipin standard (**b**). Following MS/MS of the 1,448,14 cardiolipin generated fragment ions were illustrated (**c**); cardiolipin structure is reported in the insert.

To confirm the identities of cardiolipin species, samples spectra were compared to those of a cardiolipin standard. As reported in the Figure 4, mass spectra of sample in the range of cardiolipin species (Figure 4a) were almost overlapping with those of the standard (Figure 4b). In addition, the MS/MS spectra of the ion *m*/*z* 1.448,140, was selected for fragmentation to further support the proposed assignment of cardiolipin. According to [22] the MS/MS spectra of cardiolipin [M-H]^−^ (Figure 4c) generated the following ions: two fragments at *m*/*z* 78,911 and 96,971 corresponding to phosphate and dihydrogen phosphate ions respectively; the peak at *m*/*z* 153,117 which corresponds to the neutral loss of glycerol-3-phosphate; the peak at *m*/*z* 279,496 identified as linoleic acid (C18:2), the *m*/*z* 415,515 assigned to the lyso-form of phosphatidic acid and the *m*/*z* 695,750 that corresponds to the phosphatidic acid with 18:2/18:2 fatty acyl groups.

### Fatty acid profile of CD4^+^ T cells by gas-chromatographic analysis

As a complement to MALDI-TOF analysis of phospholipids, we decided to perform a gas-chromatographic fatty acid comparative profiling of CD4^+^ T cells from MS patients and healthy subjects. Phospholipids consist of fatty acids and a significant correlation between the fatty acid composition of lymphocytes and the capacity of these cells to produce eicosanoids involved in immunoregulation has been established [23]. However, very little is known about the fatty acid composition of CD4^+^ T cells from MS patients.

As shown in Table 4, the total amount of saturated fatty acids was decreased in MS with respect to control subjects whereas both monounsaturated and polyunsaturated fatty acids were increased in MS. Among polyunsaturated fatty acids those of n-6 series were responsible for the augmented value. These findings well correlate with data obtained by MALDI-TOF demonstrating that MS-induced lipid compositional changes occur at the molecular-specie level.

**Table 4:** Fatty acid composition of CD4^+^ T lymphocytes

Fatty acids	HC	MS
14:0	1.5 ± 0.6	1.9 ± 0.7
16:0	34.3 ± 1.2	31.8 ± 2.0
16:1	0.2 ± 0.08	0.3 ± 0.09
18:0	57.2 ± 1.2	54.2 ± 4.0
18:1_n-9_	3.1 ± 0.5	5.6 ± 1.7*
18:1_n-11_	0.7 ± 0.06	0.9 ± 0.08**
18:2_n-6_	1.4 ± 0.2	2.0 ± 0.7
18:3_n-3_	0.5 ± 0.03	0.3 ± 0.01**
20:4_n-6_	1.5 ± 0.1	4.4 ± 0.2**
Σ SFA	92.9 ± 0.6	86.9 ± 4.3***
Σ MUFA	3.3 ± 0.2	5.8 ± 1.7***
Σ PUFA	3.3 ± 0.4	6.7 ± 0.9**
Total n-6	2.8 ± 0.9	6.4 ± 0.3**
SFA/PUFA	14.4 ± 1.7	7.9 ± 3.8***
UI	13.7 ± 3.2	29.1 ± 11.6***

Fatty acids were obtained from whole CD4^+^ T lymphocytes as reported in the “Methods” section. Values are expressed as percentage of total fatty acids.

*MUFA* monounsaturated fatty acids, *PUFA* polyunsaturated fatty acids, *SFA* saturated fatty acids, *UI* Unsaturation index.

Values are given as mean ± SD.

* P < 0.05; ** P < 0.01; *** P < 0.001.

## Discussion

There is growing evidence that MALDI-TOF is an excellent analytical technique for a rapid screening of lipids in biological matrices due to its high sensitivity and rapid sample preparation [20, 24]. Thus, by using this approach, individual molecular species including low-abundance phospholipid classes could be easily analyzed even in total lipid extracts [20] and directly on tissue slices without any prior step of extraction [12]. Despite all these potentialities, some limitations exist. Due to their compositional complexity, the identification of individual species at high-resolution appear to be fundamental for the unambiguous mass assignment of lipids in complex samples [25]. For this reason, high-resolution mass spectrometers have become the preferred approach for the identification and quantification of total lipid extracts in top-down lipidomic experiments [25].

In this study, we reported a direct MALDI-TOF analysis of total lipid extracts from CD4^+^ T lymphocytes using 9-AA as a matrix in negative mode. Compared to positive ion spectra, negative spectra displayed a higher amount of signals allowing the identification of several lipid species mostly phospholipids (DG, PA, PG, PE, PS, PI, CL and sphingomyelin). Moreover, the analysis of spectrum in the negative mode is facilitated by the exclusive presence of deprotonated [M-H]^−^ ion signals [26].

The interest in lipids and their analysis in the field of MS were already demonstrated by Del Boccio and colleagues [27] that showed and altered lipid pattern in the serum of MS patients. Here, for the first time, a mass-spectrometry approach was applied for the characterization of lipids from CD4^+^ T lymphocytes.

Lipids play a critical role in the structure of the central and peripheral nervous systems in particular at the cell membrane level [28]. Alterations in the phospholipid as well as in plasma membrane fatty acid composition, modifying the membrane fluidity, can affect a wide range of cellular functions such as ligand-receptor signal transduction and membrane trafficking with consequences on cell functions and survival [29]. In red blood cells from MS patients, alterations in the membrane fluidity closely correlated with inflammation and disease outcome [29]. Moreover, modifications in membrane fluidity have been reported in the central nervous system of patients with motor neuron disease [28] as well as in the brain cortex and spinal cord of patients affected by amyotrophic lateral sclerosis [30]. Despite the importance of lymphocytes in the context of MS, the lipid metabolism of these cells has received less attention. A large body of evidence indicate that CD4^+^ T cells play a role in MS pathogenesis [4], as these cells have the ability to damage and cross the blood–brain barrier, inducing axonal damage and neuronal death [6]. Lymphocytes are then important players in the onset and evolution of the disease and the main target of the current immunological therapies including interferon β reagents, glatiramer acetate, natalizumab, rituximab and Copaxone. Understanding which biological and biochemical alterations characterize lymphocytes of MS patients may be important for the identification of new biomarkers of disease and for the development of future therapies.

Our analysis by MALDI-TOF of lipid extracted from CD4^+^ T cells highlights a characteristic phospholipid pattern in MS and HC patients (Table 2). Through a multivariate statistical analysis we found several discriminant signals between the two groups (Figure 2). Among these, phospholipids in the range of 1,200–1,500 *m*/*z* were unambiguous identified as cardiolipins. However since the small sample size the potentiality of these lipids, to serve as prospective biomarkers, should be further confirmed and validated in a targeted and more extensive clinical study. In mammalian cells, cardiolipin is located almost exclusively in the inner mitochondrial membrane where it accounts for 10–20% of total mitochondrial lipids. Cardiolipin is essential for the optimal function of numerous enzymes that are involved in the mitochondrial energy metabolism [31]. Moreover, cardiolipin molecules could be also expressed on the surface of apoptotic cells and thus recognized by antiphospholipid antibodies [32]. Regarding this aspect, it has been reported that during mitochondrial stress or damage, the asymmetric cardiolipin distribution collapses resulting in its externalization to the outer mitochondrial membrane, leading to signalling events essential for mitophagy and apoptosis [33]. Interestingly, anti-cardiolipin antibodies were detected and measured in the plasma of some MS patients [34].

Several hypotheses can be considered to explain the alteration of cardiolipin observed by MALDI in MS patients. Mitochondrial dysfunction has been implicated in the development and progression of MS [35], so closely to propose mitochondrial-targeted approaches for the treatment of the disease [36]. In particular, Witte and collaborators [35] described how an enhanced density of mitochondria in MS lesions might contribute to the formation of free radicals and subsequent tissue damage. In light of our results, higher intensity of cardiolipin signal in MS patients may indicate a possible higher amount of mitochondria in these cells. The increase in the number of mitochondria may be also a strategy to prevent the decline of mitochondrial efficiency [37], as we recently observed for damaged mitochondria in cirrhotic rat livers [38].

It is well known that in most mammalian tissues cardiolipin acyl composition is predominantly comprised of 18-carbon unsaturated acyl chains, the vast majority of which is linoleic acid (C_18:2_, n-6) [33]. In our study an increased amount of cardiolipin rich in PUFA were described. PUFA are important molecules not only as structural constituent of plasma membrane, but also as mediators of inflammation. To this end, n-6 PUFA are substrates of cyclooxygenase (COX) and lipoxygenase (LOX) [39] enzymes which can convert PUFA in pro-inflammatory molecules such as prostaglandins (PG) of series 2 and leukotrinene (LT) of the series 4. An increased amount of PGE_2_ in the serum and cerebrospinal fluid of patients affected by amyotrophic lateral sclerosis was observed [40]. Moreover, LTC4, D4, E4, F4 are potent chemotactic agents contributing to neuroinflammation by enhancing vascular permeability [41]. Our gas-chromatographic analysis of CD4^+^ T cells revealed an increase in PUFA of the n-6 series as well as in the unsaturation index (UI) in MS with respect to HC. These data well correlate with the results of MALDI-TOF.

Many studies have associated the progression of pathological conditions with changes in the fatty acyl moieties of cardiolipin [22]. Shifting of fatty acids in myocardial cardiolipin from saturated fatty acids to highly unsaturated species was linked to the onset of heart failure in spontaneous hypertensive rats [42]. Moreover, we should also consider that phospholipids with saturated fatty acid side chains have been indicated as natural anti-inflammatory class of compounds that ameliorated experimental autoimmune encephalomyelitis by suppressing activation and inducing apoptosis of autoreactive T cells [43].

## Conclusion

Taken together, our data obtained by a lipidomic approach can suggest an altered mitochondrial lipid metabolism in CD4^+^ T cells from MS patients. The small number of patients represents a weakness of this preliminary study and may limit a rapid clinical application of our results. Further studies enrolling a larger cohort of patients are needed to confirm our finding and to propose a functional link about the possible role of mitochondria on the regulation of lymphocyte lipid metabolism in MS. In this context, the feasibility of MALDI-TOF/TOF in the rapid screening and characterization of lipid samples makes these results useful for potential scale-up to larger patient populations.
